# Endogenous Antimicrobial Peptide Expression in Response to Bacterial Epidermal Colonization

**DOI:** 10.3389/fimmu.2017.01637

**Published:** 2017-11-27

**Authors:** Michael Brandwein, Zvi Bentwich, Doron Steinberg

**Affiliations:** ^1^Biofilm Research Laboratory, Faculty of Dental Medicine, Hebrew University of Jerusalem, Hadassah Ein Kerem, Jerusalem, Israel; ^2^Cutaneous Microbiology Laboratory, The Skin Research Institute, Dead Sea and Arava Science Center, Masada, Israel

**Keywords:** antimicrobial peptides, dermatology, microbial immunology, cathelicidin, human beta defensins, psoriasin

## Abstract

Bacterial commensal colonization of human skin is vital for the training and maintenance of the skin’s innate and adaptive immune functions. In addition to its physical barrier against pathogen colonization, the skin expresses a variety of antimicrobial peptides (AMPs) which are expressed constitutively and induced in response to pathogenic microbial stimuli. These AMPs are differentially effective against a suite of microbial skin colonizers, including both bacterial and fungal residents of the skin. We review the breadth of microorganism-induced cutaneous AMP expression studies and their complementary findings on the efficacy of skin AMPs against different bacterial and fungal species. We suggest further directions for skin AMP research based on emerging skin microbiome knowledge in an effort to advance our understanding of the nuanced host–microbe balance on human skin. Such advances should enable the scientific community to bridge the gap between descriptive disease-state AMP studies and experimental single-species *in vitro* studies, thereby enabling research endeavors that more closely mimic the natural skin environs.

## Introduction

Human skin is the largest epithelial layer and provides a vast surface area for the interaction between the host and environmental factors (1). The skin acts as the first line of defense against physical, chemical, and biological challenges (2). The epidermis, or the outermost layer of the skin comprised mostly of stratified keratinocytes, is the first to encounter external stimuli and is therefore equipped with an arsenal of immune-modulating activities (3). Microbial colonization of the skin, long recognized as an etiological factor in many skin diseases, has been shown to induce species-specific immune responses (3, 4). Antimicrobial peptides (AMPs) are critical elements of the skin’s chemical barrier against pathogens due to their antibacterial and immunomodulatory properties (5).

Antimicrobial peptides have been discovered in a wide array of organisms and provide a first-line defense mechanism against pathogen colonization. Microbial-induced AMP expression on human skin was first reported in 1997 with the discovery of human β-defensin 2 (hBD2), a cationic peptide exhibiting broad spectrum antibiotic activity (6), in addition to cathelicidin LL-37 (7, 8). The list of skin-associated inducible AMPs has since expanded to include hBD3 (9), psoriasin (also referred to as S100A7) (10), and RNase 7 (11) in addition to the constitutively expressed dermcidin (12) and hBD1. Most AMP’s carry a cationic charge, thereby allowing them to attach to the anionic parts of the bacterial membranes. Once attached, AMP’s then take advantage of their amphipatic structure and disrupt the bacterial membrane by inserting their hydrophobic end into the bacterial membrane (13). While all of the AMPs listed above are expressed by keratinocytes, hBD2 can also be expressed by macrophages and dendritic cells, and LL-37 can be expressed by macrophages and neutrophils (14, 15). The ability of human skin to modulate bacterial colonization through the secretion of AMPs has direct consequences on the microbial landscape of the skin.

Microbial inhabitants of the skin have been of great interest as they affect skin homeostasis, and therefore are involved in important biological processes in both health and diseased states. Traditional cutaneous microbiology has focused on *Staphylococcus epidermidis, Staphylococcus aureus*, and *Propionibacterium acnes*, yet expanding experimental techniques have shown that healthy skin is a habitat for a milieu of other microorganisms, including varied species in the *Staphylococcus* genus, *Micrococcus luteus, Corynebacterium* spp., *Streptococcus mitis, Malassezia globosa, Malassezia restricta*, and others (16–18). Microorganisms immobilized on skin can display biofilm-like properties, including virulence and resistance to antibiotics (19). Importantly, biogeography, or body site, drives microbial community composition, a factor often attributed to the different glands and secretions present in moist, sebaceous, and dry areas of the skin (16, 17). Resident microorganisms have been implicated in disease pathogenesis. For example, *S. aureus* infects the skin of atopic dermatitis individuals (20) and *P. acnes* colonization is an important etiological factor in acne vulgaris (21). Interestingly, AMP expression *in situ* is also linked to diseased states. Rosacea patients secrete elevated levels of cathelicidin (22), psoriasis patients upregulate a host of AMPs (23, 24) and atopic dermatitis skin expresses less hBD and cathelicidin than that of healthy individuals (25, 26). The former two research hypotheses grew out of the observation that psoriatic individuals rarely contract skin infections (23), while atopic dermatitis patients often suffer from skin infections (27, 28).

It is evidently clear that the human body and the microbiota that colonize its skin are in a constant state of attenuation. Microbe–microbe and human–microbe interactions mediate the events that determine both the amount and type of microorganisms that reside on the skin and the results of these exchanges have broad medical and cosmetic consequences. In this review, we summarize two decades of research devoted to the antimicrobial efficacy of skin AMPs and their ability to be induced by the cutaneous microbiota. We briefly touch upon associations between AMP expression and disease states [reviewed in Ref. (29)]. Additionally, we do not focus on the signaling or alarmin mechanisms of AMP expression [reviewed in Ref. (30)], but rather their antimicrobial capabilities. We conclude with an outlook toward future AMP research, with an emphasis on integrating microbiome-era knowledge into our understanding of AMP expression.

## Experimental Models for the Assessment of AMP Expression in Human Skin

Three main experimental models can be used to detect AMP expression on human skin: *in vitro* cell lines, *ex vivo* skin fragments and *in situ* biopsies. Keratinocyte cell lines, whether primary or commercial, provide the most readily accessible and easily manipulated medium for the study of skin–microbial interactions. Keratinocytes have the distinct advantage of being able to be maintained in the laboratory for extended periods of time and their availability does not hinge upon extralaboratory sources. However, cell lines are maintained submerged in media, thereby necessitating that any microbial growth be maintained within the cell culture medium. This poses two limitations: microorganisms capable of growth on human skin may not be sustained by cell culture media and submerged growth may induce physiological changes in the microorganism that are not present when grown on the skin–air interface. Additionally, keratinocytes allow for a two-dimensional modeling of the skin surface which lacks many of the biological and physical elements present *in vivo* (31). In addition to keratinocytes, sebocyte cell lines can serve as a model substrate for bacterial-induced AMP expression. However, immortalization of sebocytes has proven difficult, and therefore, their availability as a model platform is limited.

Human skin organ cultures, typically obtained following cosmetic surgeries, serve as an additional model for cutaneous–microbial interactions. Skin explants are advantageous for the research of skin–microbe interactions for a number of reasons: they contain all of the various elements of human skin including the dermis, epidermis, and associated appendages, and unlike keratinocytes, the surface topography of skin explants matches that of human skin. Additionally, microbial growth is sustained at the skin–air interface, similar to *in vivo* conditions. Disadvantages of the *ex vivo* model include its restricted availability and limited life-span [roughly 2 weeks sustained in culture (31)]. Additionally, skin fragments are obtained from a human donor and are therefore intrinsically not sterile. They can be treated with antibiotics and antifungals before inoculating with bacteria, however, resistant bacteria may remain viable, thereby interfering with experimental procedures. Finally, bacterial immobilization on skin explants is a poorly characterized method of bacterial growth, thereby limiting the conclusions that can be garnered from such experiments.

The final human model applicable for skin–microbial interactions is biopsies from individuals in diseased states whereby the disease has a known microbial etiological factor. Such biopsies can be useful for histological analysis and molecular studies, thereby establishing associations between microorganisms and disease states. However, these specimens are often not culturable and therefore their usefulness as a model is limited. As with any model system, complementary experiments using all models are the preferred path for approaching skin–microbe research hypotheses. Specifically, the availability of keratinocytes, the wealth of knowledge available in the literature regarding keratinocyte inoculates, and the ability to manipulate experimental conditions necessitates their inclusion in an experimental setup. Validation of observations made with keratinocytes can be done *ex vivo* with the skin explant model and finally *in situ* from relevant pathological specimens.

## AMP’s and Skin Microorganisms

### *Staphylococcus* *aureus*

Skin-associated AMP expression and efficacy has been studied on a host of microorganisms, yet studies involving *S. aureus* are the most widespread (Summarized in Table 1). The first report of RNase 7 expression in human skin cells reported that challenging human primary keratinocytes with an inoculum of *S. aureus* leads to increased RNase 7 mRNA levels and that RNase 7 kills *S. aureus* in a dose-dependent manner (11). A subsequent study expanded upon these results and showed elevated RNase 7 secretion 2 h after challenging skin explants with an *S. aureus* inoculum. The study also showed that blocking RNase 7 activity in stratum corneum extracts and in skin explants hindered skin antimicrobial activity (32). Separately, anti-*S. aureus* activity of RNase 7 was shown *in vitro* (33) and secreted factors of *S. aureus* were shown to upregulate RNase 7 expression in primary keratinocytes (34).

**Table 1:** Summary of experimental reports of *Staphylococcus aureus*-induced antimicrobial peptide (AMP) expression.

		RNase 7	hBD1	hBD2	hBD3	Psoriasin	LL37
	Live	3X RNA expression (11)	Not upregulated (34)	70X RNA expression (34)	95X RNA expression (34)	Undocumented	3X RNA expression^a^ (35)
Human keratinocytes		60X RNA expression (34)	3X RNA expression^a^ (35)	2X RNA expression^a^ (35)	5X RNA expression^a^ (35)		
			Upregulated but not quantified (36)				
	
	Conditioned Media	25X RNA expression (34)	Not upregulated (34)	Not upregulated (34)	110X RNA expression (34)	Undocumented	4X RNA expression^a^ (35)
			Not upregulated (35)	3X RNA expression^a^ (35)	10X RNA expression^a^ (35)		

Human explants	Live	2X RNA expression^b^ (32)	Undocumented	Undocumented	Undocumented	Undocumented	Undocumented

Anti-*Staphylococcus aureus* activity of antimicrobial peptide		Undocumented	Effective only at high concentrations (38, 39)	Effective (38, 39)	Very effective (38, 39, 41)	Undocumented	Very effective (38, 39)

*Several studies have noted the upregulation of RNase7, hBD2, hBD3 and LL37 following challenge by S. aureus on keratinocytes. However, the AMP Psoriasin has not been studied in this model, nor have the aforementioned studies validated their results on an ex vivo model. Additionally, hBD3 and LL37 kill S. aureus at lower concentrations than the others tested*.

*^a^Ca^2+^ differentiated keratinocytes*.

*^b^Expression measured after 2 h*.

In addition to the RNase 7 observations above, human beta-defensin activity against *S. aureus* has been studied as well. Several groups have documented increased expression of hBD2 (6, 34–36) and hBD3 (34, 35, 37) in keratinocytes in response to inoculation with live *S. aureus*. Additionally, marked hBD2 and h-BD3 upregulation has been observed when inoculating keratinocytes with heat-killed *S. aureus* or *S. aureus* conditioned medium (34, 35, 37, 38). The hBD2 concentration needed to kill 100% *S. aureus in vitro* is 10 µg/ml (38) and its anti-*S. aureus* activity is increased in acidic conditions, similar to that of the skin (39). Furthermore, hBD2 works synergistically with a host of other compounds, including hBD3, lysozyme, and the serine protease Esp (39, 40). Additionally, Kisich et al. showed that constitutive, and not inducible, expression of hBD3 provides a level of clearance from *S. aureus* immediately upon infection (41).

Keratinocytes infected with *S. aureus* were shown to slightly overexpress the AMP LL-37 (38) and the anti-*S. aureus* activity of keratinocytes is partially dependent on cathelicidin expression (42). These experiments, along with the majority of the aforementioned AMP-*S. aureus* induction studies, were carried out solely on cultured keratinocytes.

### *Staphylococcus* *epidermidis*

*Staphylococcus epidermidis*, the “helpful” Staphylococci, has also been shown to induce expression of several AMPs. Percoco et al. showed that *S. epidermidis* infection significantly upregulates hBD2 and hBD3, but not Psoriasin and RNase 7, expression in skin explants (43). A further study on keratinocytes supported these results and showed that hBD2 and hBD3 expression was stimulated by *S. epidermidis* infection and that this induction is mediated through TLR2 signaling (44). Wanke et al. reported upregulation of hBD2, hBD3, and RNase 7 24 h after *S. epidermidis* colonization of keratinocytes (34), whereas both Harder et al. and Dinulos et al. examined the keratinocyte expression solely of hBD2 following *S. epidermidis* inoculation, and determined that it was upregulated in response to the commensal (6, 36). Of note, the experimental conditions for the aforementioned observations may reflect a state of infection rather than commensal living of *S. epidermidis* on the skin.

### *Propionibacterium* *acnes*

*Propionibacterium acnes*, classically regarded as a resident of the pilosebaceous unit, also has the ability to induce AMP expression. Nagy et al. showed that certain clinical strains of *P. acnes* induces hBD2 expression in keratinocytes (45). Subsequently, *P. acnes* supernatant was shown to induce the expression of hBD2 and LL-37 mRNA in keratinocytes (46). Unique among skin resident microorganisms, *P. acnes*-induced AMP expression has also been studied in sebocytes. *P. acnes* supernatant upregulated LL-37 expression in sebocytes and it was shown to work synergistically with psoriasin (47). Additionally, different strains of *P. acnes* were shown to induce hBD2 expression in sebocytes (48).

### Other Microorganisms

Various other bacteria and fungi have been shown to induce AMP expression in keratinocytes or other skin models. *Pseudomonas fluorescens* induces hBD2 and hBD3 expression in skin explants (43) and *Pseudomonas aeruginosa* can induce hBD2 and hBD3 expression in keratinocytes (6, 36). *Escherichia coli* infection of keratinocytes induces the expression of Psoriasin and of hBD2 (6, 36, 49), while *Acinetobacter baumannii* induces hBD2 and hBD3 transcripts following infection of primary skin epithelial cells (50). *Streptococcus pyogenes* induces RNase 7 expression and certain strains can induce hBD2 expression (11, 36). *Malassezia furfur* can upregulate hBD2 and hBD3 expression in human keratinocytes (51, 52). Finally, *Candida albicans* induces hBD2 expression in primary keratinocytes (6). Of additional note, the vast majority of the aforementioned experiments were carried out on cell lines, with only a select few having been performed on skin explants.

## In Vitro Efficacy of AMP in Bacterial Clearance

Following the discovery of bacterial-induced skin AMP’s, their efficacy in bacterial clearance *in vitro* was evaluated. Owing to differences in reporting standards, AMP efficacy results have been published using various different benchmarks and are therefore challenging to compare robustly to one another. An additional challenge in translating these studies are the differences between *in vivo* and *in vitro* salt concentrations and human topographical differences in salt concentrations owing to varied presence of sweat glands on the skin. Nevertheless, these studies are crucial in understanding the bacteria-modulating effects of AMPs. As per the focus of this article, we summarize AMP antimicrobial activity with regards to resident skin microorganisms exclusively.

Kisich et al., Chen et al., and Midorikawa et al. showed that LL-37 and hBD3 had significantly more anti-*S. aureus* activity than hBD1 and hBD2, and that the former two were effective in killing 99.9% of *S. aureus* in single-digit micromolar concentrations (38, 39, 41). Elsewhere, 1.6 μmol of LL-37 was reported to kill 50% of *S. aureus* (53, 54) and 50 µg/ml of LL-37 was shown to eradicate 80% of *S. aureus* (47). Ong et al. showed strong activity of LL-37 against clinical isolates of *S. aureus* from Atopic Dermatitis patients, while hBD2 was significantly less potent (25). Acidic pH sharply enhances the antibacterial capabilities of the three human beta-defensins, yet decreases that of LL-37 (39). Of particular relevance to the skin environment, hBD1, hBD2, and hBD3 are effective at low and physiologic salt concentrations (0–200 mM NaCl) (9). Additionally, AMPs often work synergistically with one another, allowing for better *S. aureus* clearance when administered/secreted in tandem (39). Of additional importance, certain AMPs can inhibit biofilm formation at subantimicrobial concentrations. For example, LL-37 inhibits 40% of *S. aureus* biofilm growth at a concentration well below its minimal inhibitory concentration (MIC) (54).

In addition, anti-*S. epidermidis* activity of skin AMP’s has been studied *in vitro*. LL-37 kills 50% of *S. epidermidis* at a concentration of 1.3 µg/ml (53). LL-37 inhibits bacterial attachment and biofilm formation of *S. epidermidis* at subinhibitory concentrations (55). *S. epidermidis* exopolysaccharide intercellular adhesin provides a level of resistance to hBD3 and LL-37 (56). hBD2 harbors anti-*S. epidermidis* activity at single-digit micromolar concentrations (36), while Psoriasin can kill *S. aureus* and *S. epidermidis* in relatively high concentrations (10).

Certain AMPs are capable of inhibiting *P. acnes* growth as well. Lee et al. reported that 50 µg/ml of LL-37 was capable of clearing 95% of *P. acnes* (47). Furthermore, single micromolar concentrations of RNase 7 eradicate *P. acnes* growth (11).

## Perspectives/Conclusion

**Figure 1:**
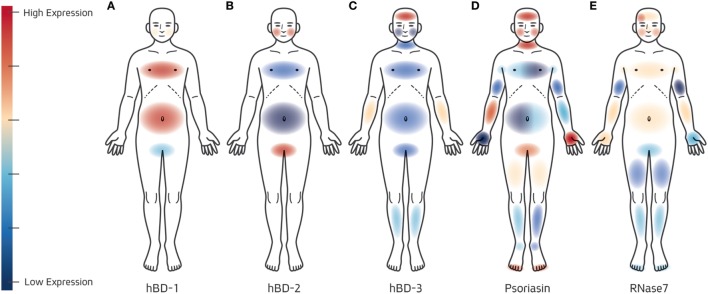
Biogeographical distribution of cutaneous antimicrobial peptide’s (AMP’s)—Gross topographical distribution of AMP expression on healthy human skin has been described by Gläser et al. (10), Falconer et al. (57), Wittersheim et al. (58), and Köten et al. (59). **(A)** hBD1 is secreted at a higher level on the abdomen and chest than elsewhere, whereas higher expression of **(B)** hBD2 and **(C)** hBD3 is reported on the cheeks and forehead, respectively. **(D)** Two studies (10, 58) examining the biogeography of Psoriasin expression reported similar results on the face, yet differences between the two studies can be seen on the palms, arms, chest, abdomen and calves (studies are represented independently on either side of the caricature). **(E)** Discrepancies between the two reports (58, 59) on RNase 7 biogeography are less dramatic, with both studies reporting higher expression on facial sites, chest, and abdomen than on arms or legs. For all examined AMPs, the variability of expression between body sites is significant, yet the biological factors leading to such expression patterns are not understood.

Given the *in vitro* and *ex vivo* inducibility of AMP expression by skin microorganisms and their ability to effectively kill bacteria and fungal residents of the skin *in vitro*, it is crucial to determine AMP expression levels and their correlation with microbial expression patterns on the skin. Several studies have already painted a general picture of the biogeographical distribution of AMPs in healthy individuals (summarized in Figure 1). hBD1 and hBD2 expression, as measured by immunohistochemistry, are generally expressed at higher levels on the scalp and plantar surface than on the axilla, abdomen, and chest (57). hBD3 is highly expressed on the forehead, and less-so in other areas of the body (58). Psoriasin is secreted mostly on the face and head, as well as in the plantar heel and palm (10, 58). RNase 7 is highly expressed on the chest, abdomen, facial sites, and forearm (58, 59). While these early descriptive studies provided pioneering confirmation that skin AMP’s were secreted in many body sites in healthy individuals and that their expression was site-dependant, further research must be done to strengthen our understanding of the AMP–microbe relationship. Microbiome studies have shown that skin bacterial and fungal communities are both site and age dependant (17, 60–62). Given the ability of specific microorganisms to induce certain AMPs, there is reason to believe that AMP expression both reflects and effects the composition of bacterial and fungal skin communities at different sites and at different ages. Tape-stripping, a non-invasive method of gathering AMPs for subsequent ELISA or western blotting quantification, could simultaneously be used to collect epidermal microorganisms for microbiome analysis. Our current research, which integrates microbial ecology profiling with AMP-specific ELISA protein quantification kits, posits that dysbiotic states are associated with an altered AMP milieu. We are specifically intrigued by diseased states with a known microbiological etiological factor, such as atopic dermatitis (dysbiosis dominated by *S. aureus*) or acne vulgaris (dysbiosis characterized by outgrowth of certain *P. acnes* strains). Such projects would be supported by recording and correlating various chemical attributes of the skin including sebum content, salinity, and moisture.

Furthermore, the aforementioned microbiome and metagenome studies have revealed a wide variety of microorganisms that are consistently found on the skin surface, yet have not received proper attention from the cutaneous microbiological community owing to their relative anonymity. Although such organisms remain understudied due to their non-pathogenic nature, their contribution toward community homeostasis and equilibrium cannot be ruled out. We therefore propose investigating their susceptibility to AMP exposure and their ability to induce AMP expression in relevant skin models.

Finally, *in vitro* studies of fungal skin residents have classically been dominated by the pathogenic fungi *M. furfur* and *C. albicans*. However, the aforementioned community-structure studies have revealed that the two species *M. restricta* and *M. globosa* comprise over 90% of the fungal skin flora. These two Malassezia species induce proinflammatory cytokine secretion following infection of keratinocytes, which is partly TLR-2 dependant, further strengthening the hypothesis that they can alter AMP expression as well (63). To the best of our knowledge, no article has addressed either the ability of skin AMPs to kill these two fungi or the ability of these fungi to induce AMP expression *in vitro, ex vivo*, or *in vivo*.

In conclusion, it is evidently clear that our skin cells are equipped with a broad arsenal of AMPs to mitigate the effects of pathogen colonization. However, our documentation of this phenomenon has largely been limited to known skin pathogens in *in vitro* models. We propose expanding the list of microorganisms studied to the myriad other bacteria and fungi that reside on our skin and emphasize the importance of validating such data on the skin explant model. Finally, we propose integrating microbiome-era knowledge into experimental design in an effort to obtain a more holistic and complete picture of skin AMP expression.

## Author Contributions

MB, ZB, and DS prepared the manuscript.

## Conflict of Interest Statement

The authors declare that the research was conducted in the absence of any commercial or financial relationships that could be construed as a potential conflict of interest.
